# First derivative ATR-FTIR spectroscopic method as a green tool for the quantitative determination of diclofenac sodium tablets

**DOI:** 10.12688/f1000research.22274.2

**Published:** 2020-04-02

**Authors:** Khairi M. S. Fahelelbom, Abdullah Saleh, Ramez Mansour, Sadik Sayed

**Affiliations:** 1 Department of Pharmaceutical Sciences, College of Pharmacy, Al Ain University, P.O. Box 64141, Al Ain, United Arab Emirates; 2 Department of Chemistry, The Hashemite University, P.O. Box 330127, Zarqa, 13133, Jordan

**Keywords:** Diclofenac sodium tablet, FTIR spectroscopy, infrared quantitative analysis, first derivative, method development

## Abstract

**Background**: Attenuated total reflection-Fourier transform infrared (ATR-FTIR) spectroscopy is a rapid quantitative method which has been applied for pharmaceutical analysis. This work describes the utility of first derivative ATR-FTIR spectroscopy in the quantitative determination of diclofenac sodium tablets.

**Methods**: This analytical quantitative technique depends on a first derivative measurement of the area of infrared bands corresponding to the CO stretching range of 1550-1605 cm ^-1^. The specificity, linearity, detection limits, precision and accuracy of the calibration curve, the infrared analysis and data manipulation were determined in order to validate the method. The statistical results were compared with other methods for the quantification of diclofenac sodium.

**Results**: The excipients in the commercial tablet preparation did not interfere with the assay. Excellent linearity was found for the drug concentrations in the range 0.2 – 1.5 w/w %.  (r ^2^= 0.9994). Precision of the method was assessed by the repeated analysis of diclofenac sodium tablets; the results obtained showed small standard deviation and relative standard deviation values, which indicates that the method is quite precise. The high percentage of recovery of diclofenac sodium tablets (99.81, 101.54 and 99.41%) demonstrate the compliance of the obtained recoveries with the pharmacopeial percent recovery. The small limit of detection and limit of quantification values (0.0528 and 0.1599 w/w %, respectively) obtained by this method indicate the high sensitivity of the method.

**Conclusions**: First derivative ATR-FTIR spectroscopy showed high accuracy and precision, is considered as nondestructive, green, low cost and rapid, and can be applied easily for the pharmaceutical quantitative determination of diclofenac sodium tablet formulations.

## Introduction

**Figure 1.**
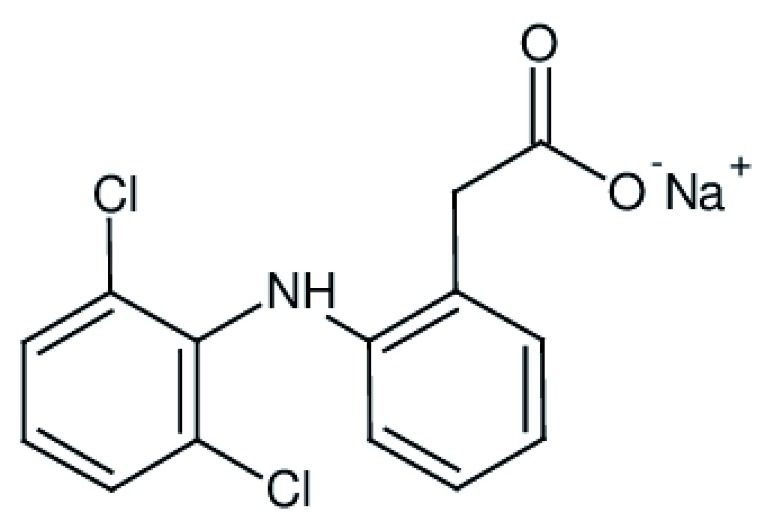
Chemical formula of diclofenac sodium.

Diclofenac sodium (DS) is a nonsteroidal anti-inflammatory drug (NSAID) that is known for its potent pharmacologic activity. It is analgesic and also ameliorates acute and subchronic inflammation. The drug plays a unique dual inhibitory role simultaneously on cyclooxygenase (COX) and lipoxygenase enzymes ^1^. The drug is widely marketed under a variety of generic and brand names and is available in several dosage forms. The chemical structure of DS is shown in Figure 1, the IUPAC nomenclature is 2-[(2,6 dichlorophenyl)aminophenyl]-acetic acid sodium salt.

Several quantitative analytical methods have been implemented to test the active pharmaceutical ingredient (API) of DS in its various marketed dosage forms. These quantitative approaches include high performance liquid chromatography (HPLC) ^2– 4^, gas chromatography ^5^, UV–visible spectrophotometry ^6^, spectrofluorometry ^7^, densitometry ^8^, potentiometry ^8, 9^, and Raman spectroscopy ^10, 11^. The HPLC methods were also employed by the official US and British pharmacopeias for the analysis of DS ^12, 13^.

However, until recently, few reports have investigated and explored the capability and feasibility of infrared spectroscopy in its quantitative approach as a potential alternative to the aforementioned classic procedures ^14^. This work aims to expand on previous reports exploring quantitative Fourier transform infrared (FTIR) approaches to quantitatively determine the APIs in a number of DS tablet dosage forms.

Traditionally, infrared spectroscopy has been extensively used in a qualitative manner. Elucidation of chemical composition has usually been achieved by analyzing incident radiation absorptions at a specific wavelength. Hence, the technique proves to be a powerful detector of functional groups. Each functional group is known to have its own distinguishable IR signature.

During the last few decades, there has been an exponential growth in the quantitative applications of IR spectroscopy ^15^.

The technique has proved to be an appealing alternative to examine DS APIs in terms of quantitative outcomes. The results of numerous reports have indicated that FTIR offers unparalleled advantages over other techniques, which makes it a versatile alternative. The technique is inherently fast and covers a wide wavenumber range. It is also non-destructive and high resolved spectra can be obtained for almost all types of samples ^16^. Simultaneous analysis of sample matrices does not necessary require special sample preparation using challenging and lengthy procedures ^17^. The technique is environmentally friendly as a result of the lack of any need for hazardous solvents or reagents ^18^. The quality of results are comparable to most powerful techniques and the cost of routine analysis is extremely economical ^19^.

Published research in this regard relies on the attenuated total reflection (ATR) sampling technique of modern FTIR spectrometers. ATR allows recording of an FTIR spectrum of solid and liquid samples directly without any further preparations ^20^. Nevertheless, the sampling of DS still requires pressing an accurately measured amount of the drug or its commercial dosage into a potassium bromide (KBr) disc to recode perfect spectra. Herein, the work retested the technique and expanded its capabilities by examining the direct spectral recording using a powder of DS (and its commercial formulations) mixed with KBr samples ^21^. The new proposal simplifies the use of the ATR-FTIR technique tremendously. The exclusion of KBr disk sampling in each run will ultimately improve the procedure in terms of the time that the overall runs require. Consequently, modern FTIR instruments are affordable and this work proves powder mixtures or thin film samples produce comparable results, implying the unnecessity of KBr discs or press machines.

The evolution of quantitative capabilities of FTIR techniques may be attributable to the vast instrumentation advancements associated with powerful computers. Additionally, sampling techniques including flow analysis (FA) and ATR have been revolutionized to allow for analysis of almost any sample type - solids, liquids, solutions, gases and vapors - directly.

Quantitative FTIR has been successfully implemented in several areas of industrial pharmacy. It has been evolving as the potential technique of quality control protocols, in particular, analysis of APIs in a broad spectrum of pharmaceutical dosage forms and formulations ^14^. The technique’s inherent characteristics and nature bears unequivocally bright prospects. It is considered a purely green analytical chemistry technique. It is fast, easy to operate by a moderately experienced technician, can analyze any sample with little to no preparation, covers a wide range of spectrums to analyze most pharmaceutical products, has high resolution and is nondestructive. Importantly, it is environmentally friendly since no solvent or harmful reagents are required for the complete analysis ^22^.

This work also demonstrates and utilizes modern spectrometers’ powerful function of the first derivative spectra. The function provided the basic tool that allows the spectrometer resolution to be tuned to the degree required to greatly enhance the band separation of each spectrum. In this work, the ATR-FTIR spectrum of the pure DS was recorded. The powerful data processing and acquisition software associated with the spectrometer allowed the first derivative spectra to be obtained. The first derivative spectra indicated the degree of IR band overlapping within each spectrum. The selection of the IR band that best correlates with the concentration of DS without any interference of other bands is based solely on the first derivative spectra.

## Methods

### Materials and reagents

IR spectroscopic-grade potassium bromide was checked prior to usage through the loss on drying method based on the recommended British Pharmacopia (BP) procedure ^13^, which involves introducing 1g of the sample into a dry bottle, reheating and reweighing until a constant weight is obtained.

Reference DS chemical standard was provided by Neopharma Pharmaceutical Manufacturing, Abu Dhabi, UAE. Three different DS commercial tablets were obtained from local pharmacies in Al Ain city, UAE, namely: Rumafen (diclofenac sodium 50 mg); Olfen TM 50 (diclofenac sodium 50 mg); and Diclogesic 50 (diclofenac sodium 50 mg).

### Equipment

The FTIR instrument IRAffinity-1 CE (Shimadzu®, Kyoto, Japan), equipped with MiracleTM Single Reflection Horizontal ATR Accessory (Pike® Technologies) and IResolution Software (Shimadzu, version 1.60; Parameters: Measurement mode: % transmittance; Apodization: Happ-Genzel; No of scans: 15; Resolution: 16.0; Range (cm ^-1^): Min 700, Max 2000), was used for the screening and quantitative analysis in this study. Non-proprietary spreadsheet processing software could be used to analyze the raw data obtained. The samples were dried in an oven (WiseVen®, Won-032, S. Korea) and an analytical balance (AUW220D, Shimadzu®) was used for all weights.

### Calibration curve

A calibration curve was prepared from six different DS standard concentrations within the range of 0.2–1.0 % w/w. An appropriate quantity of DS was diluted with potassium bromide to get each concentration and was thoroughly ground in a mortar for 10 min to ensure sample homogeneity. Infrared spectra of each measurement were converted to the first derivative spectra. The area under the curve (AUC) of each calibration standard was measured in the range 1550–1605 cm ^-1^. This band corresponds to the CO stretching of carboxylic sodium salts.

### Quantitative determination of diclofenac sodium tablets

To determine the API content for the commercial tablets in this study, ten tablets were accurately and individually weighed, then the whole sample was finely powdered for each brand. Samples were prepared by mixing and thoroughly grinding an appropriate quantity of each tablet powder with potassium bromide to get 0.5% w/w of DS. 100 mg of the mixture was transferred to the diamond ATR top plate of the spectrometer over the diamond crystal. IR measurements were run in triplicate and the average was calculated for each run.

### Data analysis

SPSS version 21 (SPSS Inc., Chicago, IL, USA) was used for descriptive and analytical statistics.

## Results and discussion

### Method validation

The proposed ART-FTIR analytical method was validated according to the International Council for Harmonization of Technical Requirements for Pharmaceuticals for Human Use guidelines ^23^.

Validation of the method included the following parameters: linearity, selectivity, limit of detection (LOD), limit of quantification (LOQ), accuracy, precision and robustness.

### Linearity

The proportional relationship between the AUC and concentration was evaluated by constructing a linear regression of five concentrations (0.2, 0.4, 0.6, 0.8, 1%). The excellent linearity obtained was indicated by the correlation coefficient value r ^2^ = 0.9994. Table 1 shows the data analysis of the DS calibration curve and the linear relationship is presented in Figure 3a and 3b.

**Table 1.** Data analysis of diclofenac sodium calibration curve.

Wavelength (cm ^-1^)	Concentration range (% w/w)	Regression equation	R2	LOD (%)	LOQ (%)
1550-1605	0.2 – 1.5	Y = 1.375X -0.014	0.9994	0.0528	0.1599

LOD, limit of detection; LOQ, limit of quantification.

### Selectivity

**Figure 2.**
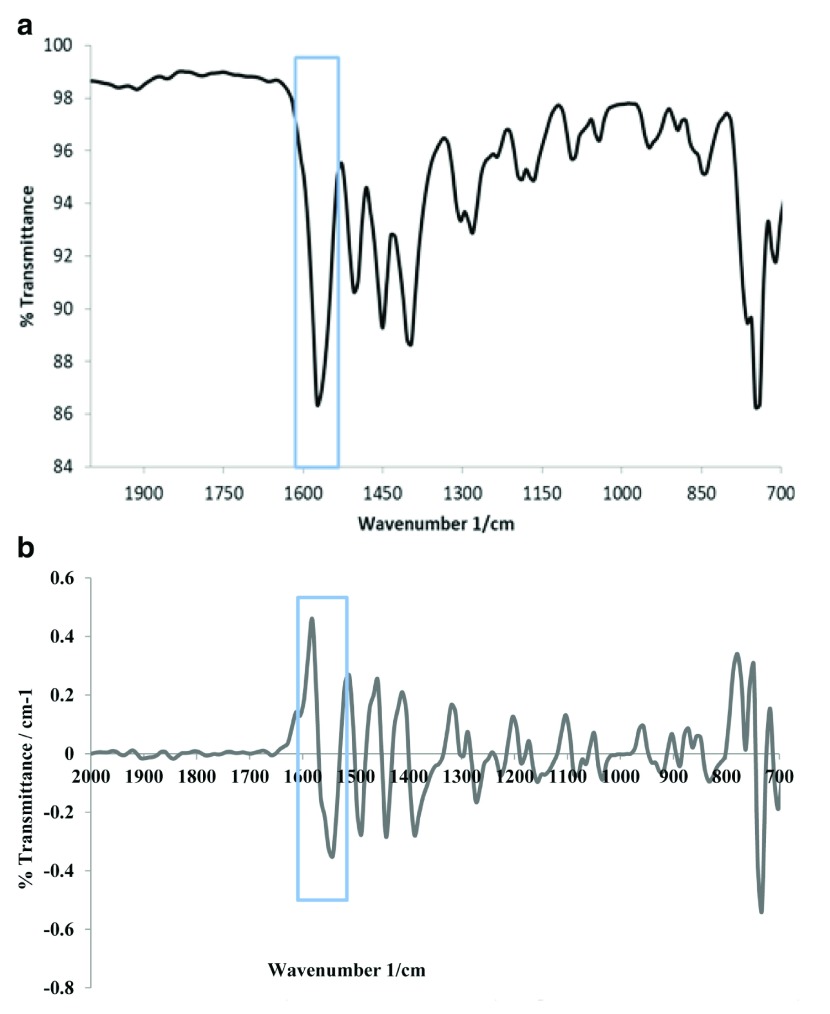
( **a**) Spectrum of pure diclofenac sodium mixed with potassium bromide (KBr; 0.6% w/w). ( **b**) First derivative spectrum of pure diclofenac sodium mixed with KBr (0.6% w/w).

**Figure 3.**
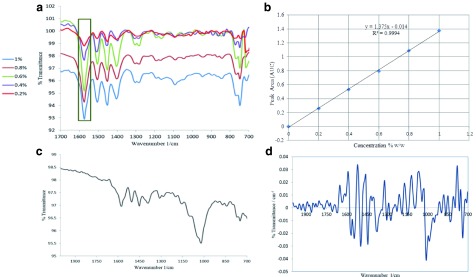
( **a**) Overlay spectra of different diclofenac sodium reference substance concentrations. ( **b**) Calibration curve of diclofenac sodium showing the linear regression equation. ( **c**) Direct Fourier transform infrared (FTIR) spectra of Olfen Tablet. ( **d**) First derivative FTIR spectra of Olfen Tablet.

Selectivity is used to justify the ability of the method to accurately quantify the existence of DS in the presence of other pharmaceutical additives. The selectivity of our method was verified by comparing diclofenac tablets to pure diclofenac. Bands used for quantification were only unique to diclofenac. Figure 2a and 2b represents the direct and first derivative of ART-FTIR spectra, which indicate the CO high absorption band at 1550–1605 cm ^-1^. The first derivative shows a clear band without any overlapping from the other peaks. Additionally, Figure 3c and 3d represents the direct and first derivative spectra of Olfen tablet, respectively. The obtained data represented in these figures showed no interference from the excipients and additives present in the tablet formulation.

### LOD and LOQ

The LOD is the lowest amount of analyte at which that an analyte can be detected. While the LOQ is the lowest amount of analyte at which an analyte can be measured accurately and precisely.

LOD=3.3σ/S(Equation 1)

LOQ=10σ/S(Equation 2)

Where σ is the standard deviation and S is the slope.

By applying Equation 1 and Equation 2, the obtained values of LOD and LOQ were 0.052757% and 0.159869%, respectively ( Table 1). The low values for both parameters indicate the high sensitivity of this study ^24^.

### Accuracy

The accuracy of the method was evaluated using the calibration method. Three different brands (A, B, and C) of diclofenac tablets were analyzed. 10 tablets of each brand were crushed to a fine powder and mixed with KBr to prepare 0.5% w/w diclofenac/KBr. Mean recovery of Tablets A, B and C was calculated to be 99.8%, 101.5% and 99.4, respectively ( Table 2). The obtained results are in agreement with USP and BP recovery range for DS tablets ^12, 13^.

**Table 2.** FTIR quantitative analysis of different DS tablet dosage forms.

Samples of tablet dosage forms	DS-API content % w/w	Recovered DS-API % w/w	% Recovery
Sample (A) Diclogesic 50 mg	0.511	0.505	98.82
	0.511	0.508	99.41
	0.511	0.517	101.17
	**Average**		**99.81**
Sample (B) Olfen 50 mg (B)	0.5	0.499	99.80
	0.5	0.517	102.80
	0.5	0.510	102.00
	**Average**		**101.54**
Sample (C) Rumafen 50 mg (C)	0.514	0.505	98.24
	0.514	0.511	99.41
	0.511	0.514	100.58
	**Average**		**99.41**

FTIR, Fourier transform infrared; DS, Diclofenac sodium; API, Active pharmaceutical ingredient.

### Precision and robustness

The precision of the method was assessed by repeatability and intermediate precision studies. Repeatability and robustness analysis were performed by analyzing the 0.5% w/w (mixed with KBr) peak areas (AUC) of each brand. Three readings for each sample were recorded and analyzed in one day (for the intra-day precision) and two readings were recorded on two consecutive days (for the inter-day precision). The results were very precise, as represented by the standard deviations, which ranged between 0.008343 and 0.020255, while the percent relative standard deviation (% RSD) was between 1.26095 and 3.03781.

## Conclusion

The proposed first derivative ATR-FTIR spectroscopic method is considered as a green, nondestructive, low cost, fast, sensitive, accurate and precise technique for the quantitative analysis of DS in its pure and tablet dosage formulation and can be easily applied for quantitative determination and quality control.

## Data availability

### Underlying data

Harvard Dataverse: Utility of ATR-FTIR Spectroscopic Method as Green Tool for Pharmaceutical Analysis of Diclofenac Sodium Tablets. https://doi.org/10.7910/DVN/6SJZ7W ^25^.

Data are available under the terms of the Creative Commons Zero “No rights reserved” data waiver (CC0 1.0 Public domain dedication).
