# Supplementary material for: First derivative ATR-FTIR spectroscopic method as a green tool for the quantitative determination of diclofenac sodium tablets
Source: F1000Res. 2020 Apr 2;9:176. Originally published 2020 Mar 10. [Version 2] doi: 10.12688/f1000research.22274.2 (PMC7096217; doi:10.12688/f1000research.22274.2)
Supplement: Peer review history

# Peer review: Reviewer response for version 2

**DOI:** 10.5256/f1000research.25346.r61922

**Published:** 2020-04-27

**Authors:** Mokhtar M. Mabrouk

**Affiliations:** 1 Department of Pharmaceutical Analytical Chemistry, Faculty of Pharmacy, Tanta University, Tanta, Egypt

This article describes first derivative ATR-FTIR spectroscopic method as a green tool for quantitative determination of the non-steroidal anti-inflammatory drug diclofenac sodium in its tablet dosage form. The proposed validated method is a new green analytical technique as it avoids use of organic solvents or tedious extraction methods. The article expresses all the necessary scientific parameters for quantitative analysis. The procedure can be applied simply for other similar applications. The simplicity and low cost of the technique offer advantages over other analytical methods in the area of drug analysis. Statistical evaluation of data are sufficient and represents clearly the precision as well as accuracy.

Is the rationale for developing the new method (or application) clearly explained?

Yes

Is the description of the method technically sound?

Partly

Are the conclusions about the method and its performance adequately supported by the findings presented in the article?

Yes

If any results are presented, are all the source data underlying the results available to ensure full reproducibility?

Yes

Are sufficient details provided to allow replication of the method development and its use by others?

Yes

Reviewer Expertise:

Pharmaceutical Analytical Chemistry, Drug analysis and Pharmaceutical chemistry.

I confirm that I have read this submission and believe that I have an appropriate level of expertise to confirm that it is of an acceptable scientific standard.

# Peer review: Reviewer response for version 2

**DOI:** 10.5256/f1000research.25346.r61921

**Published:** 2020-04-07

**Authors:** Baker Jawabrah Al-Hourani

**Affiliations:** 1 Department of Biology and Chemistry, Embry-Riddle Aeronautical University, Prescott, AZ, USA

All suggested changes have been made properly. Thank you!

Is the rationale for developing the new method (or application) clearly explained?

Yes

Is the description of the method technically sound?

Yes

Are the conclusions about the method and its performance adequately supported by the findings presented in the article?

Yes

If any results are presented, are all the source data underlying the results available to ensure full reproducibility?

Yes

Are sufficient details provided to allow replication of the method development and its use by others?

Yes

Reviewer Expertise:

Drug design and Discovery Research.

I confirm that I have read this submission and believe that I have an appropriate level of expertise to confirm that it is of an acceptable scientific standard.

# Peer review: Reviewer response for version 2

**DOI:** 10.5256/f1000research.25346.r61920

**Published:** 2020-04-02

**Authors:** Yasser Fakhri Mustafa, Hasan Aldewachi

**Affiliations:** 1 Department of Pharmaceutical Chemistry, College of Pharmacy, University of Mosul, Mosul, Iraq

The authors have replied to all the reviewers concerns and made all the necessary amendments.

Is the rationale for developing the new method (or application) clearly explained?

Yes

Is the description of the method technically sound?

Yes

Are the conclusions about the method and its performance adequately supported by the findings presented in the article?

Yes

If any results are presented, are all the source data underlying the results available to ensure full reproducibility?

Yes

Are sufficient details provided to allow replication of the method development and its use by others?

Yes

Reviewer Expertise:

Medicinal Chemistry, Chemistry of natural products, Pharmaceutical analysis, Organic synthesis, and instrumentations.

We confirm that we have read this submission and believe that we have an appropriate level of expertise to confirm that it is of an acceptable scientific standard.

# Peer review: Reviewer response for version 1

**DOI:** 10.5256/f1000research.24569.r61173

**Published:** 2020-03-19

**Authors:** Baker Jawabrah Al-Hourani

**Affiliations:** 1 Department of Biology and Chemistry, Embry-Riddle Aeronautical University, Prescott, AZ, USA

Quantitative determination of diclofenac sodium in tablet dosage was carried out by using the ATRFTIR device. This is a unique methodology and considered as a part of the green chemistry field. The work is clearly presented, reproducible, clean, and full of important information, which can be used by any third party.

 First comment is regarding a typo in the first paragraph in the introduction, line 5: Please change “cyclo-oxygenase” to “cyclooxygenase”.

 Second comment is regarding the scientific content of the same paragraph, line 5: Please specify the type of the cyclooxygenase: 1/2 or both. The same thing regarding the lipoxygenase enzyme.

Is the rationale for developing the new method (or application) clearly explained?

Yes

Is the description of the method technically sound?

Yes

Are the conclusions about the method and its performance adequately supported by the findings presented in the article?

Yes

If any results are presented, are all the source data underlying the results available to ensure full reproducibility?

Yes

Are sufficient details provided to allow replication of the method development and its use by others?

Yes

Reviewer Expertise:

Drug design and Discovery Research.

I confirm that I have read this submission and believe that I have an appropriate level of expertise to confirm that it is of an acceptable scientific standard.

# Response

**Published:** 2020-03-20

**Authors:** Khairi Salem

**Authors Response for Comments of reviewers 1 - Yasser Fakhri Mustafa and Hasan Aldewach:**

1. All data in Table 2 is represented in % w/w. Sample B data in the Table 2 was corrected according to the reviewers comments in order to be consistent with samples A and C.
2. The standard deviations (SD) and relative standard deviations (RSD) have been summarized under precision and robustness section.
3. This work describes the Utility of ATR-FTIR for the analysis of uncoated tablet of diclofenac sodium, as the excipients is not interfering in the analysis of active ingredient we expect that the coating material may need certain extraction process.

**Authors Responses for Comments of reviewer 2 Baker Jawabrah Al-Hourani:**

DS is known to be nonselective COX inhibitor. The inhibition of COX-1 (constitutive isoform) is assumed to be the main cause of undesirable side effects of the drug. However; the inhibition effects of the COX-2 (inducible isoform) yield the desired effects. The exceptional potency of DS among other NSAIDs was explained on the basis of the additional 5-lipoxygenase (5-LOX) indirect  inhibitory effect of the drug.

Reference

Fiorucci S, Meli R, Bucci M, Cirino G, Dual inhibitors of cyclooxygenase and 5-lipoxygenase. A new avenue in anti-inflammatory therapy, Biochemical Pharmacology. 2002; 62(11):1433-8, DOI: 10.1016/S0006-2952(01)00747-X

Author : Khairi M.S. Fahelelbom

# Peer review: Reviewer response for version 1

**DOI:** 10.5256/f1000research.24569.r61176

**Published:** 2020-03-16

**Authors:** Yasser Fakhri Mustafa, Hasan Aldewachi

**Affiliations:** 1 Department of Pharmaceutical Chemistry, College of Pharmacy, University of Mosul, Mosul, Iraq

This study aimed to investigate one of the green analytical chemistry tools for quantitative determination of Diclofenac sodium (DS) in tablet dosage forms by using ATR-FTIR technique. The novelty of this work arises from excluding the need for sample preparation like the using of KBr discs. I find the work interesting, informative, and worthy to be indexed. However I have some concerns/suggestions after carefully reading the report.

1. 2 ^nd^ column in Table 2 needs consistency in the expression of concentration of DS because sample B is expressed in terms of %, while samples A&C were expressed as quantities.
2. I would rather suggest expressing the standard deviation (SD)and relative standard deviation (RSD) in a table as follows:
  Sample name        SD              RSD
  Sample A        0.0083             1.26%
  Sample B
  Sample C
3. Would the authors be able to state whether this method suits coated tablet forms of DS?

Is the rationale for developing the new method (or application) clearly explained?

Yes

Is the description of the method technically sound?

Yes

Are the conclusions about the method and its performance adequately supported by the findings presented in the article?

Yes

If any results are presented, are all the source data underlying the results available to ensure full reproducibility?

Yes

Are sufficient details provided to allow replication of the method development and its use by others?

Yes

Reviewer Expertise:

Medicinal Chemistry, Chemistry of natural products, Pharmaceutical analysis, Organic synthesis, and instrumentations.

We confirm that we have read this submission and believe that we have an appropriate level of expertise to confirm that it is of an acceptable scientific standard.
